# Effects of Miosis on the Visual Acuity Space under Varying Conditions of Contrast and Ambient Luminance in Presbyopia

**DOI:** 10.3390/jcm13051209

**Published:** 2024-02-21

**Authors:** Maksymilian Onyszkiewicz, Julian Hilmers, Robert Rejdak, Eberhart Zrenner, Torsten Straßer

**Affiliations:** 1 Institute for Ophthalmic Research, University of Tuebingen, 72076 Tuebingen, Germany; maksymilian.onyszkiewicz@gmail.com (M.O.); ezrenner@uni-tuebingen.de (E.Z.); 2 Chair and Department of General and Pediatric Ophthalmology, Medical University of Lublin, 20-059 Lublin, Poland; 3 STZ Eyetrial, University Eye Hospital Tuebingen, 72076 Tuebingen, Germany; robert.rejdak@umlub.pl; 4 University Eye Hospital Tuebingen, 72076 Tuebingen, Germany

**Keywords:** visual acuity, VA-CAL, pilocarpine, presbyopia, miosis, pupil, depth of focus

## Abstract

**Background:** Presbyopia is an age-related ocular condition, typically affecting individuals aged over 40 years, characterized by a gradual and irreversible decline in the eye’s ability to focus on nearby objects. Correction methods for presbyopia encompass the use of corrective lenses, surgical interventions (corneal or lens based), and, more recently, the FDA-approved topical administration of 1.25% pilocarpine. While prior research has demonstrated the efficacy of daily pilocarpine eye drop application in enhancing near visual acuity by increasing the depth of focus leveraging the pinhole effect, limited knowledge exists regarding its influence on visual acuity under varying conditions of contrast and ambient luminance. **Methods:** This study aims to investigate the impact of these variables on visual acuity, employing the VA-CAL test, among 11 emmetropic and 11 presbyopic volunteers who reported subjective difficulties with near vision. This study includes evaluations under natural conditions with a pinhole occluder (diameter of 2 mm), and subsequent administration of 1% pilocarpine (Pilomann, Bausch + Lomb, Laval, Canada). **Results:** The VA-CAL results demonstrate the expected, statistically significant effects of contrast and ambient luminance on visual acuity in both emmetropic and presbyopic volunteers. Furthermore, in emmetropic individuals, the application of pilocarpine resulted in a statistically significant reduction in visual acuity. In contrast, presbyopes did not exhibit statistically significant differences in the visual acuity space under either the pinhole or pilocarpine conditions when compared to natural conditions. **Conclusions:** The pharmacological treatment of presbyopia with pilocarpine eye drops, intended to enhance near vision, does not adversely affect visual acuity in presbyopes. This suggests that pilocarpine may offer a viable alternative for individuals averse to wearing corrective eyewear.

## 1. Introduction

Presbyopia is an age-related, progressive, and irreversible decline in the eye’s accommodative ability, typically initiating around the age of 40 [1]. This condition impairs the eye’s capability to effectively focus on nearby objects, thereby affecting daily activities and decreasing the quality of life. Accumulating evidence suggests that presbyopia is caused by age-related changes in lenticular structures, mainly due to the loss of lens elasticity [2,3]. Croft et al. provide an extensive introduction to the mechanisms underlying accommodation and presbyopia [4].

Several studies imply that in 2015, over 1.8 billion people were suffering from presbyopia, and the prevalence is expected to peak at approximately 2.1 billion in 2030 [5,6]. Access to corrective measures that restore near vision is limited in some parts of the world. It is estimated that reading glasses are available only for 6–45% of patients living in developing countries. The high prevalence of uncorrected presbyopia is linked to a lack of affordable treatment and adequate diagnosis [7,8,9,10,11]. Conversely, the broad accessibility of corrective aids, such as reading glasses in developed countries, could mean that potential alternative presbyopic treatment options are often overlooked [12]. Methods to correct presbyopia include corrective lenses or glasses, but also corneal- or lens-based surgery [5,13,14,15,16,17,18]. Furthermore, recent data imply that lens softeners, such as Novartis’ investigational presbyopia correcting drop UNR844, could restore some degree of lens elasticity, allowing for better accommodation [19]. When the lens regains elasticity, the eye can focus better on close distances. Accommodation occurs through the central displacement of cytosolic proteins in the fibers of the eye lens. Aging is linked with oxidative stress. It leads to the formation of disulfide bonds between lens proteins, resulting in impaired cytosolic flow, lens stiffening, and presbyopia. Lipoic acid, a prodrug that hydrolyzes disulfide bonds in its active form, could potentially lead to a softer eye lens, improve its elasticity, and as a final effect improve the focus on close distances [19].

It has long been known that near vision improves by natural constriction of the pupils through increased illumination [20]. Thereby, the depth of field (DOF) of the eye increases as the diameter of the pupil decreases [21,22]. This effect can be leveraged to help people with presbyopia to see adequately over a greater range of object distances [22,23].

The pinhole effect created by a small pupil blocks distorted and unfocused light rays and isolates more focused central and paracentral rays through the central aperture, thereby reducing aberrations of the optical system as a whole and enhancing image quality and visual acuity [24,25].

A simple approach to leverage the pinhole effect for increasing DOF and thereby improving the near visual acuity of presbyopes is pinhole glasses. It was shown that these glasses improve uncorrected distance as well as near visual acuity [26,27,28], and reduce the required accommodative power by about 15% [26]. However, the use of pinhole glasses results in decreased visual quality, such as an impaired visual field, reduced reading speed, and lower contrast sensitivity [27,28]. Furthermore, a shortened tear break-up time was observed [28], probably leading to a worsening of subjective ophthalmic symptoms [28], uncomfortable feelings, and excessive eye fatigue [26].

Alternative approaches, aiming to reduce the problem of visual field restriction, are artificial pupils integrated into contact lenses, corneal inlays, or intra-ocular lenses (IOLs). Here, vignetting effects are reduced, since the artificial pupil is much closer to the natural pupil [29]. A scleral contact lens with a pinhole aperture and an opaque periphery was first developed by Ziller [29] and was further pursued by several other groups in the following decades [23,30,31]. The same optical principle has been applied in corneal inlays [32,33] and IOLs [34]. At present, commercially available options include one corneal inlay, the AcuFocus KAMRA^TM^ inlay [35,36], and two IOLs, namely the AcuFocus IC-8 [37,38] and the Xtrafocus from Morcher GmbH [39,40]. In contrast to contact lenses, both corneal inlay and IOL avoid the problem of decentration of the aperture [29]. For a thorough survey of small aperture optics employed in the treatment of presbyopia and the various approaches to creating a pinhole effect, see Charman [29].

Several researchers have revisited the original concepts of leveraging DOF as an aid for presbyopia. The eye possesses an inherent aperture, the pupil, which, upon constriction, extends the DOF, thereby potentially enhancing near visual acuity. Rather than introducing an artificial pupil, a pharmacologically induced miosis can be employed to achieve the same result. While originally described almost half a century ago [23], the use of miotic drugs has drawn some attention in recent years [41,42]. For an overview of currently used drugs for inducing miosis see the comprehensive reviews of Renna et al. [43] and Karanfil and Turgut [44]. 

The most commonly used drug for inducing pupil constriction is pilocarpine, a cholinergic muscarinic receptor agonist that acts through the M3 muscarinic receptors. Pilocarpine binds and activates muscarinic M3 receptors located on the iris sphincter [45] and on the ciliary body, inducing both pupillary miosis and, potentially, accommodative spasm [46]. Moreover, pilocarpine stimulates the contraction of the longitudinal ciliary muscle fibers, which pull on tendons terminating in the trabecular meshwork and the inner wall of Schlemm’s canal. Pilocarpine is used routinely in ophthalmology, especially for glaucoma therapy to stimulate pupils’ constriction, thereby increasing the outflow of aqueous humor [47].

In 2021, the U.S. Food and Drug Administration approved AGN-190584 (Vuity^TM^, Allergan/AbbVie, North Chicago, IL, USA), a 1.25% pilocarpine hydrochloride (HCl) solution, as the first commercially available eye drops for treating presbyopia based on the results of two phase 3 clinical studies, GEMINI 1 [48] and GEMINI 2 (ClinicalTrials.gov identifier: NCT03857542). The manufacturers reported that 31% of individuals aged between 40 and 55 experienced an improvement of three-or-more lines in corrected near visual acuity under mesopic conditions, without a loss of more than one line in corrected distance visual acuity [48]. The results of the GEMINI 1 and 2 clinical trials showed adverse reactions to the use of pilocarpine solution. About 15% of participants experienced headaches. Other side effects were mostly mild to moderate. Some researchers suggest combining miotics with other drugs, for example, aceclidine with tropicamide. Aceclidine is a muscarinic agonist, weaker than pilocarpine, and tropicamide has an antimuscarinic effect that allows for dilation without significantly affecting accommodation. Furthermore, the combination of pilocarpine with oxymetazoline, an alpha-adrenergic agonist that causes vasoconstriction and mydriasis, could reduce hyperemia but also reduce depth of field due to the effect on pupil size [49].

All approaches using the small-aperture optics principle for increasing the DOF and thus improving near vision, whether non-invasive, invasive, or pharmacological, have in common that the amount of light reaching the retina is reduced and therefore a darker image is produced [23,29]. Particularly at low-luminance and low-contrast conditions, this results in a loss of resolution [50,51] and distance visual acuity [52,53]. This increases the risk of accidents and falls, especially in invasive and pharmacologically induced miosis because the pupils are prevented from opening wide in the dark. Poor twilight vision is particularly dangerous when driving or operating machinery. These concerns are likely to be greater in phakic presbyopes, whose ocular transmissibility is already significantly reduced compared to young adults, mainly due to greater absorption in the crystalline lens. Lenticular light loss increases slowly in early presbyopia but tends to increase rapidly after about the age of 60 with the onset of early cataract development, although there is considerable individual variation [54,55].

Accordingly, Vuity’s prescribing information states ‘Patients should be advised to exercise caution in night driving and other hazardous occupations in poor illumination’ [46].

The purpose of this study was to investigate the effect of an artificially or pharmacologically induced pinhole aperture on visual acuity under varying contrast and ambient luminance conditions.

Standard visual acuity testing, with luminance between 80 and 320 cd/m^2^, a maximum optotype contrast, and contrast vision testing, performed separately in clinical routine, do not represent the range of luminance and contrast conditions present in everyday life and thus are unable to assess the full range of visual performance. Therefore, the VA-CAL test, which determines visual acuity as a function of contrast and ambient luminance, was developed as an alternative test procedure to enable a realistic determination of the visual acuity space [56].

In a recent study, we used the VA-CAL test to show that short-wavelength cutoff filter glasses can improve visual acuity by approximately 0.6 logMAR in individuals with achromatopsia, who are extremely sensitive to glare, especially in high-ambient-light and low-contrast conditions [57]. In contrast, in this study, we specifically expect a reduction in visual acuity under conditions of low ambient light and low contrast. The results may help to set out recommendations for the possibilities and limitations of pharmacologically induced miosis as a treatment for presbyopia.

## 2. Materials and Methods

This study complies with the Declaration of Helsinki and is approved by the Institutional Review Board of the Medical Faculty of the University in Tuebingen (734/2022BO2).

### 2.1. Participants

A sample size of at least 20 participants (10 per group) was estimated to detect a statistically significant change in VA (α = 0.05, β-1 = 0.95, mean difference 0.3 ± 0.15 logMAR) using G*Power [58,59], based on previous studies [56,57].

Volunteers were recruited from the staff of the Centre for Ophthalmology at the University of Tuebingen according to the inclusion and exclusion criteria listed in Table 1.

After signing informed consent, an initial ophthalmic examination was performed, followed by measuring the best-corrected visual acuity (BCVA) using a Snellen chart and examination of the anterior eye segment with a slit lamp.

### 2.2. General Examinations

Before and about 30 min after the installation of pilocarpine eye drops, each participant’s pupil diameter and amplitude of accommodation were assessed using the push-up method by employing a RAF rule with Duane’s line figure for determining the near point [60]. Furthermore, post-experiment measurements of intraocular pressure were obtained.

### 2.3. VA-CAL Procedure

Landolt C rings were presented as randomly rotated in 45° steps at ambient luminances (AL) of 0, 30, 320, 3000, 5000, and 10,000 cd/m^2^ NS at Weber contrasts of 10%, 30%, and 70%. The participants were seated at a 1 m distance to the screen with their heads stabilized in a combined chin-and-head rest and instructed to indicate the opening direction of the Landolt C ring using a wireless keypad within a maximum of 10 s. Misses were considered incorrect. Visual acuity was determined using a four-alternative forced-choice (4AFC) QUEST adaptive staircase routine [61]. The detailed setup and procedure have been described previously [56,57].

The test was carried out monocularly (eye with better VA or leading eye) without pupil dilation using the same best correction of refractive error as ascertained in BCVA and was repeated for each of the three conditions, i.e., with best-corrected naked eye (corrected for the test distance), with 2 mm pinhole occlude in a trial frame, and 30 min after application of commercially available 1% pilocarpine eye drops (Pilomann, Bausch + Lomb, Laval, QC, Canada).

### 2.4. Statistics

The effect of the refractive status and the application of pilocarpine on near point and pupil diameter were analyzed using linear mixed-effects models with the fixed factors: group (control, presbyope) and time point (pre-, post-experiment), as well as their interaction, and the subject as a random effect to account for repeated measures and missing data. The models were fitted using restricted maximum likelihood (REML). The variance inflation factors (VIF) of the predictors were calculated and assuredly fell well below the common threshold value, indicating no collinearity between them [62]. The residuals were visually confirmed to follow a normal distribution and the homogeneity of the variances was ensured using the Brown–Forsythe test and reported in case of violations [63,64]. Post hoc comparisons of the least-squares means using two-tailed *t*-tests were conducted in the case of statistically significant effects.

To investigate the hypotheses regarding the effects of pilocarpine or a pinhole occluder on achievable visual acuity under different levels of contrast and ambient luminance, a full-factorial weighted linear mixed-effects model was employed, with the fixed factors being group (control, presbyopia) and condition (naked eye, pinhole, pilocarpine); continuous contrast level (0.1, 0.3, 0.7) as a quadratic term; and continuous ambient luminance (0, 30, 320, 3000, 5000, 10,000) modeled as 4-knotted spline to account for a non-linear relationship with the dependent variable, visual acuity [65,66]. The subject was treated as a random effect. The model was fitted using REML. To address the unequal variance in visual acuity across the groups and conditions, weights were calculated using a two-step procedure [67,68,69]: Firstly, a log variance model was fitted using the independent variables age and condition to the residuals of a fit of the unweighted original model. Secondly, the normalized reciprocal predictions of the log variance model were employed as weights for the weighted linear mixed-effects model.

Subsequently, least-squares means were analyzed using pairwise multiple comparisons with Dunnett’s C test, which takes into consideration unequal variances [70,71]. If not otherwise stated, an alpha level of 0.05 was used for all statistical analyses.

All statistical analyses were performed using JMP Pro 17 (SAS Institute, Cary, NA, USA).

## 3. Results

### 3.1. Participants’ Demographics

Eleven emmetropic (age 22–35 years, median 28 years, three women) and eleven presbyopic (age 45–68 years, median 60 years, six women) volunteers were included in the study according to the inclusion and exclusion criteria listed in Table 1.

**Figure 1:**
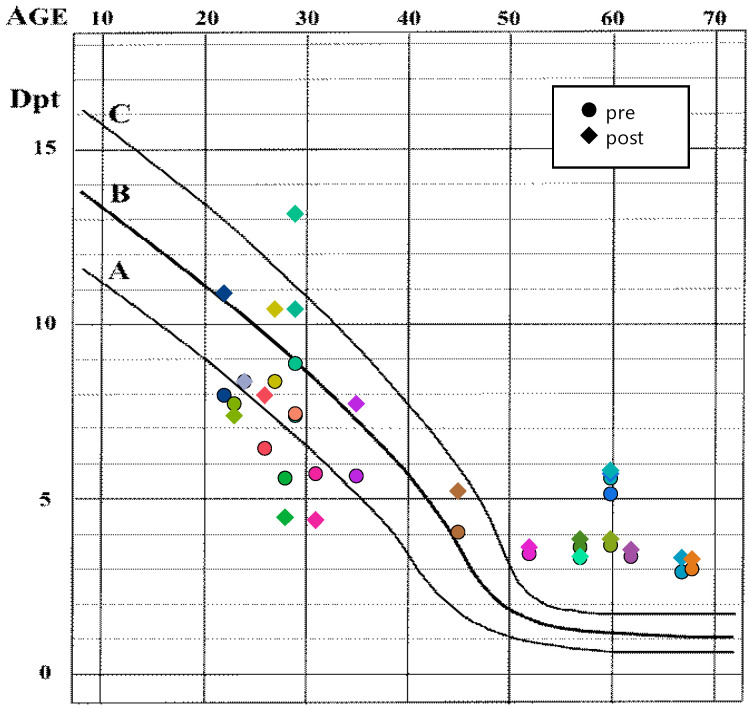
Accommodation amplitude as a function of the age of the participants overlaid over the classical Duane curve [1] (mean (**B**) and approximate upper (**A**) and lower (**C**) standard deviation are shown) before (circles) and after (diamonds) the experiment (i.e., after application of pilocarpine). The colors indicate the single participants, and the symbols pre- and post-experiment measurements. Modified from: Hans Strasburger, CC BY-SA 4.0.

Figure 1 depicts the accommodation amplitude of the participants as a function of age before and following the experiment, i.e., after the application of pilocarpine eye drops. The accommodation amplitudes follow the classical Duane curve [1]. The average pupil diameter prior to the experiment was 3.3 ± 0.2 mm (mean ± SD), independent of the age of the participants (Table 2).

### 3.2. Effects of Refractive State and Time Point on Accommodation and Pupil Diameter

The linear mixed-effects models revealed the statistically significant effects of the refractive state (group) and the time point (pre- and post-treatment) on the maximally achievable near point of accommodation and the statistically significant effect of the time point on the pupil diameter (Table 2). Interestingly, no statistically significant effect of group on the pupil diameter was found. It should be noted that the pupil diameter models’ residuals exhibited heteroscedasticity (Brown–Forsythe test: *F*(1, 41) = 4.3650, *p* = 0.0375).

**Figure 2:**
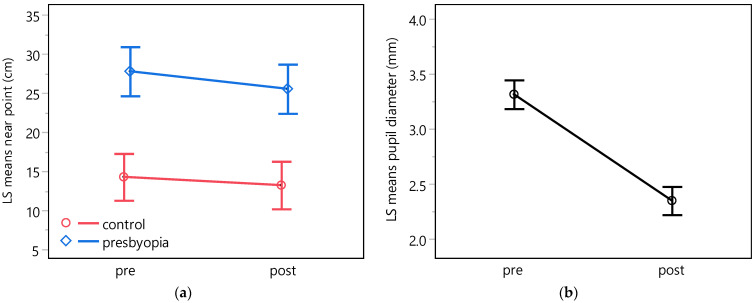
Least-squares means for the near point distance (**a**) and the pupil diameter (**b**) obtained from the linear mixed-effects models with fixed factors group, time point, and their interaction. The near point distance exhibits statistically significant effects for both group and time point, whereas the pupil diameter is solely influenced by the time point. Notably, the interaction between group and time point did not reach statistical significance in either model. Whiskers indicate the 95% confidence interval.

Post hoc comparisons conducted using paired *t*-tests of the least-squares means of the near point distances between emmetropic controls and presbyopic participants demonstrated an expected statistically significant smaller near point distance for emmetropic compared to presbyopic participants, with a mean difference of −12.9 cm (equivalent to 3.6 D). Furthermore, a small but statistically significant difference of −1.0 cm (equivalent to 0.4 D) was observed between the least-squares means near points measured before and after the experiment, irrespective of the participants’ refractive state (Figure 2a, Table 3). 

The corresponding analysis of the pupil diameter revealed a statistically significant reduction of 1.0 mm in the pupil diameter after the experiment, independent of the refractive state (Figure 2b, Table 3).

### 3.3. Effects of Pinhole Occluder and Pilocarpine on Visual Acuity at Varying Levels of Contrast and Ambient Luminance in Presbyopes and Emmetropic Controls

The weighted linear mixed-effects model revealed the statistically significant effects of ambient luminance and contrast on visual acuity. Additionally, statistically significant effects were found for the factor condition (naked eye, pinhole occlude, pilocarpine) as well as for the interactions of group × condition and group × condition × ambient luminance (Table 4).

**Figure 3:**
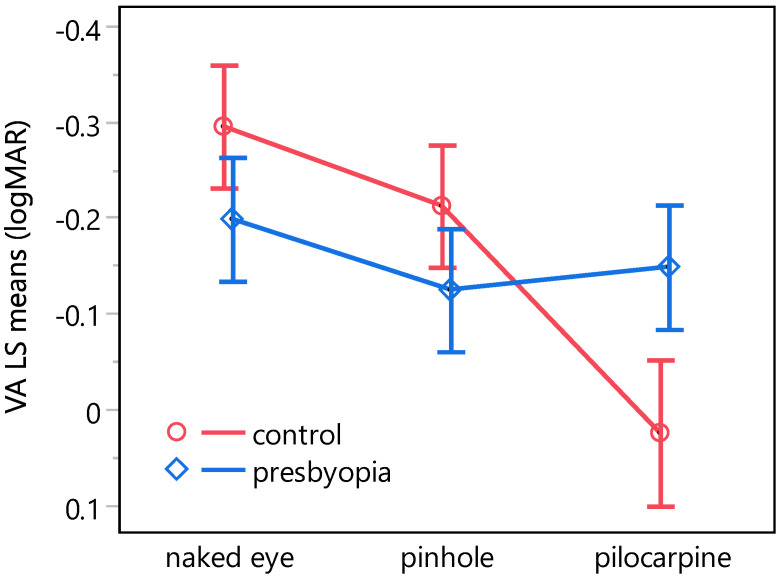
Least-squares means plot of the result of the weighted linear mixed-effects model for the visual acuity dependent variable and the interaction of the fixed effects condition and group. Error bars represent 95% confidence intervals of the mean.

Figure 3 illustrates the least-squares means of visual acuity as a function of both group and condition. The post hoc Dunnett’s C test revealed statistically significant reductions in visual acuity within the control group, exhibiting −0.32 logMAR and −0.21 logMAR for the pilocarpine condition in comparison to the naked eye condition and the pinhole condition, respectively. In contrast, within the presbyopia group, no statistically significant differences were observed between the pinhole and the pilocarpine condition when compared to the naked eye condition. Table 5 lists the differences across all combinations resulting from the interaction between group and condition.

**Figure 4:**
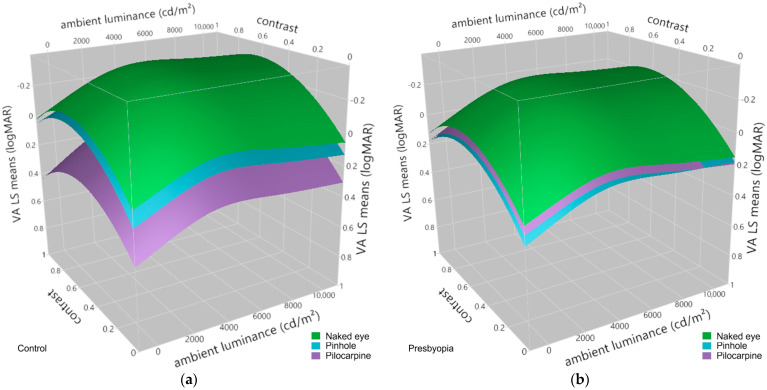
Visual acuity space predicted as least-squares means from the weighted linear mixed-effects model as a function of contrast and ambient luminance for (**a**) the control group and (**b**) the presbyopic group. The colors of the surfaces represent the different conditions: naked eye (green), pinhole (cyan), and pilocarpine (purple).

Figure 4 illustrates the effects of the pinhole occluder and pilocarpine eye drops, respectively, on visual acuity in emmetropes and presbyopes at different levels of contrast and ambient luminance in comparison to the naked eye condition as predicted from the linear mixed-effects model.

## 4. Discussion

The encouraging increases in life expectancy seen in many parts of the world in recent decades have led to a growing proportion of elderly individuals. Unfortunately, this has been accompanied by an increase in the prevalence of presbyopia, in which the eye lens loses its flexibility as part of the natural aging process, resulting in a loss of the eye’s ability to accommodate nearby objects [1]. Since no method has yet been found to reduce this inexorable process, presbyopes need some sort of correction to see objects at close distances clearly [10]. Untreated visual impairment caused by presbyopia reduces the quality of life by affecting social interactions, hobbies, and daily activities: while near vision is essential for activities such as reading or using a smartphone, intermediate vision is required for computer work, cooking, and social interactions [72,73,74,75,76].

In addition to the first-described uses of glass lenses to correct presbyopia in the late 13th century [77], the utilization of the pinhole effect to enhance the depth of field in vision has been recognized for many centuries [29,78] and is recommended as a treatment for a wide variety of refractive disorders up to today [78,79,80]. However, by the 1970s, stenopeic glasses were considered obsolete and became less popular, most likely due to issues related to reduced light and peripheral vision as well as aesthetic reasons [29]. Interestingly, the concept of pinholes has been revived with the development of several non-surgical and surgical procedures intending to create artificial pupils using contact lenses [23,29,30,31], corneal inlays [32,33], or intra-ocular lenses [34]. Nevertheless, none of the available approaches could match the effectiveness of physiological accommodation in providing high-contrast, sharp images at different distances [15,16] and suffer from limitations such as problems with night vision, double vision, difficulties with contrast sensitivity, halos, glare, ghost images, or corneal scaring [42,81,82]. Chang et al. classify the currently available options for presbyopia treatment in view of expanding functional through focus, a term they coined to describe the ability to see at all distances with minimal latency [83].

Rather than artificially creating the pinhole effect, miotics have recently regained attention as a potential treatment option for presbyopia. Pupillary miosis can be achieved by stimulating the iris sphincter muscle or inhibiting the dilator. The most potent miotic agents are parasympathomimetic drugs activating the parasympathetic pathway [43,44], with pilocarpine as the most widely used agent. Pilocarpine is used routinely in ophthalmology, especially in glaucoma therapy [47]. It is a cholinergic muscarinic receptor agonist that acts by binding and activating the M3 muscarinic receptors on the iris sphincter [45], resulting in pupil constriction. While originally described almost half-a-century ago [23], the use of miotic drugs as treatment for presbyopia has drawn some attention in recent years [41,42], and in 2021, the U.S. Food and Drug Administration approved Vuity^TM^ (Allergan/AbbVie), a 1.25% pilocarpine hydrochloride (HCl) solution, as the first commercially available eye drops for treating presbyopia. The pharmacological approach uses the eye’s iris to create a small aperture that blocks aberrant rays from reaching the retina, and ultimately increases the depth of field and the clarity of retinal images [84]. The opening is in the plane of the pupil, which avoids excessive restriction of the field of view [29]. The quality is sufficient for many users for simple close-up work that does not require particularly clear vision [48]. However, after application, the amount of light entering the eye is reduced due to the narrow pupil, causing impaired twilight vision. In addition, pilocarpine can lead to contraction of the ciliary muscle, which may on the one hand improve accommodation and in turn near vision, but on the other hand can impact visual acuity [85]. In rare cases, pilocarpine could result in an accommodative spasm [46]. Nevertheless, safety studies have shown that pilocarpine in concentrations up to 1.5% has an acceptable safety and tolerability profile [49].

Varying contrasts and ambient luminance conditions affect visual acuity within a natural scene [28,86]. Typically, a combination of aberration and diffraction, both varying with the pupil diameter, define the retinal image quality and hence visual acuity. Under natural conditions, the eye compensates for the effects of diffraction and aberration by adjusting pupil diameter [50,87,88,89], a mechanism that is absent in pharmacologically induced miosis.

To quantify the effect of miosis on visual acuity at different levels of contrast and ambient luminance, a pinhole effect was induced artificially and pharmacologically using pinhole glasses and 1% pilocarpine eye drops (Pilomann, Bausch + Lomb, Laval, Canada), respectively.

The subjects’ pupil diameter showed a statistically significant reduction of 1.0 mm (95% confidence interval: [1.1, 0.8] mm) on average after pilocarpine eye drop installation (Figure 2b, Table 3), which corresponds to the reduction reported by Waring et al. [48] and Price et al. [49]. The baseline pupil diameter of 3.3 mm is consistent with previously published values [90]. Neither the refractive group nor its interaction with the time point had a statistically significant effect on pupil diameter (Table 2), although a decrease with age is usually observed [90,91,92]. However, at intermediate luminance, the difference between age groups is small [90]. It should be noted that the residuals of the pupil diameter model exhibited heteroscedasticity, resulting in an underestimation of the variance, biased confidence intervals, and smaller *p*-values. However, linear mixed-effects models are known to be robust to violations of distributional assumptions [93].

Interestingly, the amplitude of accommodation in the emmetropic group follows Duane’s curve distribution, while in the presbyopic group, it is, by an average of 3.7 D, better than expected (between 1 and 1.5 D, Figure 1) [1]. After the administration of pilocarpine, the mean improvement in maximum near point distance was 1.6 cm (95% confidence interval: [0.3, 3.0] cm), equivalent to approximately 0.39 D, as shown in Table 3. This improvement was observed independently of the refractive group, as indicated by Table 2 and Figure 2a. The improvement in near point distance following pilocarpine-induced miosis was relatively slight, particularly considering that the increased depth of focus (DoF) should alleviate blurriness, which is utilized in determining the amplitude of accommodation [94]. The pupil diameter was probably too large to create a noteworthy boost in the depth of field. According to research by Charman and Whitefoot, the effects are negligible unless the pupil diameter is less than 2 mm. The DoF is highly variable between individuals [95] and depends on viewing conditions such as object luminance [96]. Furthermore, prolonged refractive history may cause blur adaptation [97], which may partly contribute to the improvement in near visual acuity in reading letters, as found by Waring et al. [48]. For an in-depth review regarding DoF and the measurement of the amplitude of accommodation, refer to Burns et al. [94]. For a review of the possible sources of errors in the measurement of the amplitude of accommodation, see Burns et al. [98].

In accordance with previous research [56,99,100], a linear mixed-effects model revealed the statistically significant effects of ambient luminance and contrast on visual acuity. Interestingly, the refractive state (group: emmetrope, presbyope) was found to have no statistically significant effect alone but its interaction with the condition (naked eye, pinhole, pilocarpine) yielded statistically significant results (Table 4). A post hoc Dunnett’s C test (Table 5), conducted to investigate the source of this effect in the presence of heteroscedasticity, revealed that it occurred mainly from differences in visual acuity between the comparisons of interactions involving the emmetropic control and the presbyopic group. Notably, no statistically significant changes in visual acuity were observed between the naked eye condition and either the pilocarpine or the pinhole condition in the presbyopic group. A higher concentration of pilocarpine might result in a larger difference; however, several studies have shown that concentrations of 1.0% and 1.5% pilocarpine provoke a similar mean gain in a mesopic, high-contrast UNVA letter test of 5 letters [49,53]. Conversely, within the emmetropic group, the use of pilocarpine appears to result in a statistically significant deterioration in visual acuity of 0.32 logMAR compared to the naked eye condition. However, this difference is most likely due to an accommodative spasm experienced by a single young emmetropic subject following the administration of the pilocarpine eye drops, a known side effect of pilocarpine [101], resulting in transitory myopia [102]. This adverse reaction makes the drug almost unusable in the younger age group [101,103].

## 5. Conclusions

Pharmacologically induced miosis, specifically through the use of pilocarpine eye drops, has demonstrated minimal impact on visual acuity, even under conditions of low contrast and reduced luminance. This suggests that pharmacological interventions utilizing pilocarpine to enhance near vision could represent a viable alternative for individuals averse to wearing glasses, offering a convenient and inconspicuous option. While patient preferences may vary, a less conspicuous therapeutic approach, devoid of visible indicators of age-related visual decline, presents optometrists with an alternative tool in the management of presbyopia. However, it is imperative to acknowledge that the long-term effects of daily application of low-dose pilocarpine eye drops warrant further investigation [104]. The results of this study raise the interesting question of whether the improvement in visual acuity achieved through the induction of the pinhole effect by miosis could be realized by simpler, non-pharmacological means, such as adequate bright lighting and the use of high-contrast black text on white paper instead.

## Figures and Tables

**Table 1:** Summary of the in- and exclusion criteria of the study.

	Inclusion Criteria	Exclusion Criteria
Emmetropes	Agreement to participate in the study Age ≤ 35 years No suspected or confirmed eye disease (self-reported) Uncorrected monocular visual acuity ≤0.1 LogMAR Spherical equivalent < ± 0.5 D	Incapable of giving consent Age < 18 years Uncorrected monocular visual acuity >0.1 LogMAR Spherical equivalent ≥ ±0.5 D Any eye disease influencing the endpoint according to the judgment of the investigator and study physician
Presbyopes	Agreement to participate in the study Age ≥ 40 years Subjective complaints of poor near vision that impact activities of daily living BCVA ≤ 0.1 logMAR No suspected or confirmed eye disease (anamnesis)	Incapable of giving consent Age < 40 years BCVA > 0.1 logMAR Any IOL implant, iritis, asthma, or any eye disease influencing the endpoint according to the judgment of the investigator and study physician

**Table 2:** Effects of group (control, presbyope) and time point (pre-, post-experiment) on near point and pupil diameter analyzed using linear mixed-effects models.

Fixed Effects and Interaction	Near Point (n = 41, R^2^_adj_. = 0.97)		Pupil Diameter (n = 43, R^2^_adj_. = 0.89)	
	*F*-Statistic	*p*-Value ^1^	*F*-Statistic	*p*-Value ^1^
group	*F*(1, 19.13) = 41.32	<0.0001 ***	*F*(1, 20.28) = 0.39	0.5399
time point	*F*(1, 18.29) = 6.86	0.0172 *	*F*(1, 19.86) = 189.51	<0.0001 ***
group × time point	*F*(1, 18.29) = 0.88	0.3593	*F*(1, 19.86) = 0.54	0.4692

^1^ Alpha level = 0.05; asterisks indicate the level of significance: * *p* < 0.05, ** *p* < 0.01, *** *p* < 0.001.

**Table 3:** Results of post hoc comparisons using paired *t*-tests of the least-squares means obtained from the linear mixed-effects models.

Comparison (LS Means ± SE)		Diff.	95% CI	*t*-Value	*p*-Value ^1^
Near point (cm)					
control (13.7 ± 1.4)	presbyope (26.7 ± 1.5)	12.9	[8.7, 17.1]	6.53	<0.0001 ***
pre (21.0 ± 1.0)	post (19.4 ± 1.1)	−1.6	[−3.0, −0.3]	−2.62	0.0172 *
Pupil diameter (mm)					
pre (3.3 ± 0.1)	post (2.3 ± 0.1)	−1.0	[−1.1, −0.8]	−13.77	<0.0001 ***

^1^ Alpha level = 0.05; asterisks indicate the level of significance: * *p* < 0.05, ** *p* < 0.01, *** *p* < 0.001.

**Table 4:** Results of the linear mixed-effects model with the dependent variable visual acuity.

Fixed Effects and Interactions ^1^	*F*-Statistic	*p*-Value ^2^
group	*F*(1, 21.06) = 0.01	0.9304
condition	*F*(2, 1141.10) = 143.40	<0.0001 ***
ambient luminance	*F*(3, 1140.92) = 142.88	<0.0001 ***
contrast	*F*(1, 1140.92) = 472.61	<0.0001 ***
contrast × contrast	*F*(1, 1140.92) = 158.77	<0.0001 ***
group × condition	*F*(2, 1141.10) = 56.19	<0.0001 ***
group × contrast	*F*(1, 1140.92) = 0.21	0.6456
group × ambient luminance	*F*(1, 1140.92) = 0.05	0.8193
condition × contrast	*F*(2, 1140.92) = 1.50	0.2251
condition × ambient luminance	*F*(2, 1140.92) = 1.27	0.2801
contrast × ambient luminance	*F*(1, 1140.92) = 2.66	0.1034
group × condition × contrast	*F*(2, 1140.92) = 0.14	0.8662
group × condition × ambient luminance	*F*(2, 1140.92) = 3.85	0.0215 *
group × contrast × ambient luminance	*F*(1, 1140.92) = 0.00	0.9770
condition × contrast × ambient luminance	*F*(2, 1140.92) = 0.94	0.3917
group × condition × contrast × ambient luminance	*F*(2, 1140.92) = 0.19	0.8252

^1^ n = 1188, R^2^_adj_. = 0.7462. ^2^ Alpha level = 0.05; asterisks indicate the level of significance: * *p* < 0.05, ** *p* < 0.01, *** *p* < 0.001.

**Table 5:** Results of the Dunnett’s C post hoc test comparing least-squares means differences of the interaction between group and condition.

Comparison		VA LS Means ± SE (logMAR)		Diff. [95% CI] ^1^ (logMAR)
control, naked eye	control, pinhole	−0.30 ± 0.03	−0.21 ± 0.03	−0.08 [−0.21, 0.04]
	control, pilocarpine		0.02 ± 0.04	−0.32 [−0.46, −0.18] *
	presbyopia, naked eye		−0.20 ± 0.03	−0.10 [−0.22, 0.03]
	presbyopia, pinhole		−0.12 ± 0.03	−0.17 [−0.30, −0.04] *
	presbyopia, pilocarpine		−0.15 ± 0.03	−0.15 [−0.27, −0.02] *
control, pinhole	control, pilocarpine	−0.21 ± 0.03	0.02 ± 0.04	−0.24 [−0.38, −0.10] *
	presbyopia, naked eye		−0.20 ± 0.03	−0.01 [−0.14, 0.11]
	presbyopia, pinhole		−0.12 ± 0.03	−0.09 [−0.21, 0.04]
	presbyopia, pilocarpine		−0.15 ± 0.03	−0.06 [−0.19, 0.06]
control, pilocarpine	presbyopia, naked eye	0.02 ± 0.04	−0.20 ± 0.03	0.22 [0.08, 0.36] *
	presbyopia, pinhole		−0.12 ± 0.03	0.15 [0.01, 0.29] *
	presbyopia, pilocarpine		−0.15 ± 0.03	0.17 [0.03, 0.32] *
presbyopia, naked eye	presbyopia, pinhole	−0.20 ± 0.03	−0.12 ± 0.03	−0.07 [−0.20, 0.05]
	presbyopia, pilocarpine		−0.15 ± 0.03	−0.05 [−0.18, 0.08]
presbyopia, pinhole	presbyopia, pilocarpine	−0.12 ± 0.03	−0.15 ± 0.03	0.02 [−0.10, 0.15]

^1^ The asterisk (*) denotes a statistically significant difference as determined by the Dunnett’s C test (i.e., the 95% confidence interval does not encompass 0).

## Data Availability

The data presented in this study are available on request from the corresponding author.
